# Unraveling the difference in aroma characteristics of tomato flesh with different colors using HS-SPME-GC–MS/MS and *E*-nose combined with multivariate data analysis

**DOI:** 10.1016/j.fochx.2026.103594

**Published:** 2026-01-30

**Authors:** Junrong Xu, Yushi Lu, Jing Cui, Yunzhi Liu, Wenjin Yu, Changxia Li

**Affiliations:** College of Agriculture, Guangxi University, Nanning 530004, China

**Keywords:** Volatile organic compounds, Color-indicative compounds, Machine learning algorithms, Flavor evaluation, Relative odor activity value

## Abstract

Aroma profiles of tomato flesh from 16 tomato varieties with 4 different fruit colors were characterized by headspace-solid phase microextraction coupled with gas chromatography-triple quadrupole mass spectrometry (HS-SPM*E*-GC–MS/MS) and electronic nose (*E*-nose). A total of 154 volatile organic compounds (VOCs) were qualitatively and semi-quantitatively identified, including 29 aldehydes, 22 hydrocarbons, 21 alcohols, 32 unknown compounds, and others, with aldehydes being the most abundant. Twenty-six characteristic VOCs might be major contributors to tomato flavor by relative odor activity value analysis. The correlation between *E*-nose sensor responses and GC–MS/MS volatile profiles was also examined. The machine learning models were constructed to show potential for distinguishing the fruit colors of the tomato. Finally, 16 fruit color-indicating VOCs were selected via multivariate data analysis. The present study will contribute to flavor evaluation and provide a chemical basis for future tomato flavor improvement, to be accompanied by sensory validation.

## Highlights

• HS-SPME-GC–MS/MS identified 154 VOCs in the flesh of 16 tomato varieties.
• Twenty-six characteristic volatiles contributed to various aroma notes.
• *E*-nose and GC–MS/MS combined with LightGBM showed potential to classify fruit colors.
• Multivariate data analysis uncovered 16 fruit color-indicative VOCs.

## 1 Introduction

Tomato (*Solanum lycopersicum* L.) is a climacteric fruit whose flavor is jointly determined by soluble sugars, organic acids, and various volatile organic compounds (VOCs) (Ramadoss et al., 2025). The levels of sugars and acids directly affect the sweetness and acidity of fruit, while the composition of VOCs confers their unique flavor characteristics (Xiao et al., 2024). Over the past few decades, breeders have primarily focused on developing tomato varieties with high yields, disease resistance, improved storage and transportability, often at the expense of flavor, resulting in a degradation of tomato flavor (Wang et al., 2022). Therefore, the study of aroma patterns of different tomato varieties is currently receiving increasing attention from breeders.

Generally, the biosynthetic pathways of VOCs are classified into three major categories: the fatty acid metabolism pathway, the amino acid metabolism pathway, and the terpenoid pathway (Xu et al., 2023). For example, during tomato ripening, β-ionone, synthesized by the carotenoid pathway (part of the terpenoid pathway), contributes to floral and woody aromas (Xu et al., 2023). Additionally, damascenone, pseudoionone, and geranylacetone are the sources of sweet and green aromas (Distefano et al., 2022). In immature tomatoes, high levels of methyl salicylate (via the amino acid pathway) repel herbivores, yet consumers generally dislike its spicy flavor (Distefano et al., 2022). Both 6-methyl-5-hepten-2-one and α-citral, synthesized via the terpenoid pathway, exhibit positive correlations with lycopene and are recognized as key contributors to the fresh fruity smell of tomatoes (Rambla et al., 2014). In addition, α-citral has also been shown to enhance sweetness in mature tomato fruits (Tieman et al., 2012). C_6_ volatiles (such as (Z)-3-hexenal, hexanal, 1-hexanol, and (E)-2-hexenal) and C_5_ volatiles (such as 1-penten-3-one), synthesized via the fatty acid pathway, are considered the most abundant volatile compounds in tomatoes (Rambla et al., 2014). However, these volatiles contribute little to the overall tomato flavor. The amino acid pathway produces compounds such as 2-isobutylthiazole, phenylethanol, 3-methylbutanal, 3-methylbutanol, and phenylacetaldehyde in tomato fruits (Wang et al., 2017). Overall, these VOCs interact with each other to further enrich the aroma complexity of the tomato.

The unique aroma of tomato is produced by a complex blend of numerous aromatic compounds, rather than a simple combination of one or a few volatiles. Thus, it is necessary to accurately and completely extract and analyze the volatile profiles of the tomato. The method of headspace solid-phase microextraction (HS-SPME) provides the advantages of good reproducibility, easy operation, solvent-free, and time efficiency compared to conventional extraction techniques (Raza et al., 2019). It is commonly combined with gas chromatography–mass spectrometry (GC–MS) and widely applied for the extraction and analysis of volatiles in tomatoes (Zhang, Ye, et al., 2024), pears (Feng et al., 2024), Chinese chives (Xie et al., 2022), and other fruits and vegetables. Additionally, the electronic nose (*E*-nose) system is an innovative instrument designed to simulate the human olfactory system. It operates using an electrochemical sensor array to detect odor signals and generate a smell fingerprint (Li, Wang, et al., 2024). Although GC–MS and *E*-nose provide valuable information on aroma characteristics, their data are often high-dimensional and multivariate; thus, algorithmic approaches are required for interpretation, for example, chemometrics (Wang et al., 2025), machine learning (Wen et al., 2023), and deep learning (Zhang, Liu, et al., 2024). To date, few studies have investigated tomato flavor by combining *E*-nose and GC–MS data with machine learning. Moreover, the aroma profiles of tomatoes are mostly focused on different types of cultivars or different tissues, and have not been fully explored for tomatoes with different fruit colors.

The present study aimed to comprehensively characterize the discrepancies of VOC profiles in tomato flesh from 16 tomato varieties with different fruit colors, including both commercial and heirloom varieties, using HS-SPME combined with gas chromatography-triple quadrupole mass spectrometry (HS-SPM*E*-GC–MS/MS) and *E*-nose. Furthermore, the characteristic VOCs of tomato were established by relative odor activity values (rOAVs). The flavor fingerprint profiles were constructed using rOAVs and *E*-nose data. Lastly, multiple machine learning classification algorithms were performed to explore the potential for distinguishing among color varieties. The fruit color-indicative volatile compounds were ascertained using multivariate statistical analysis. These findings will support instrumental flavor evaluation, sensory validation, and the selection of tomato varieties.

## 2 Materials and methods

### 2.1 Plant materials

**Fig. 1:**
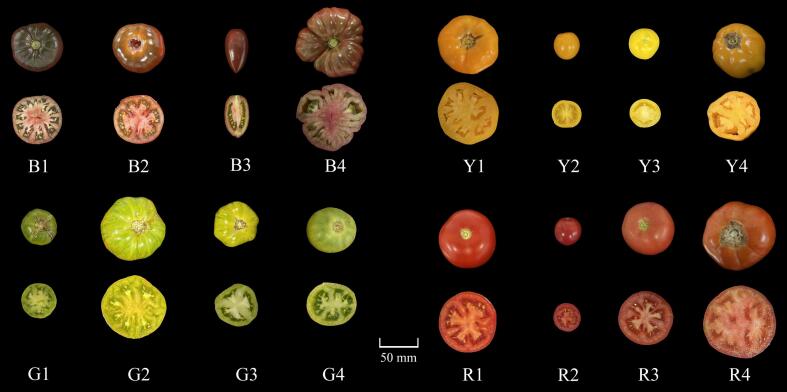
Appearance of 16 tomato varieties grouped by fruit colors (brown, yellow, green, and red). (For interpretation of the references to color in this figure legend, the reader is referred to the web version of this article.)

Sixteen tomato varieties with four fruit colors (brown, yellow, green, and red) were used as materials in this experiment; their information was presented in Table S1, including code, fruit color, fruit size, variety type, maturity, and source. In addition, given the wide genetic and phenotypic diversity of cultivated tomatoes, comprehensive aroma-related studies have not been conducted on all varieties used in this study. The tomato plants were cultivated in the greenhouse of Guangxi University (22.85°N, 108.29°E) from September 2023 to February 2024. All experimental materials were grown under the same environment and managed uniformly during the growing stage. We picked the ripe fruits from the third ear on all tomato plants, and their appearances were exhibited in Fig. 1. Fruit colors were determined by both visual assessment and colorimetric measurement. A SC-10 portable chroma meter (Guangdong THREENH Technology Co., China) was used to measure lightness (L*), greenness/redness (a*), blueness/yellowness (b*), and hue angle (H°) values of tomatoes, following the method referred to (Li, Hou, et al., 2024). The distributions of L*, a*, b*, and H° values were presented in Fig. S1. These tomato fruits were free of disease and mechanical damage and were uniform in size and color for each variety (Zhang et al., 2023). Additionally, we harvested each variety on the same day and ensured that the selection of fruits had the same number of days from flowering to harvest and had achieved optimal color and brightness. Each tomato variety was sampled in three biological replicates with 40 fruits per replicate. The surface of the fruits was cleaned, the flesh was collected, the samples were mixed and immediately immersed in liquid nitrogen, and finally stored in a − 80 °C refrigerator. The seeds and locular gel were removed to improve sample uniformity and ensure better comparability.

### 2.2 Extraction of VOCs by HS-SPME

According to the methods of Ballard et al. (2023) and Cortina et al. (2018) with some modifications, HS-SPME was performed. The sample (10 g) was ground into powder with liquid nitrogen and collected into a 50 mL brown headspace vial. Then, a magnetic stirring rotor, 1 mL EDTA/NaOH (pH = 7.5), 10 mL saturated CaCl_2_ solution, and 10 μL of 81.9 mg·L^−1^ 2-octanol were added to the vial. In particular, 2-octanol was the internal standard and was diluted to 81.9 mg·L^−1^ with chromatographic-grade methanol. We immediately screwed the headspace vial with a PTFE‑silicon gasket and mixed adequately by an ultrasonic cleaner. Sample vials were sonicated for 1 min and then equilibrated at 50 °C at 1000 rpm for 10 min. Subsequently, we inserted 50/30 μm DVB/CAR/PDMS (Supelco Inc., Bellefonte, PA, USA) fiber pre-activated at 250 °C for 30 min into the sample vial with heating and stirring continuously for 40 min. After extraction, the fiber was immediately transferred to the GC–MS/MS injection port to desorb at 250 °C for 5 min. We conducted three biological replicates for each tomato variety.

### 2.3 Detection of VOCs by GC–MS/MS

The isolation and characterization of VOCs in tomato was carried out on Thermo Trace1300 gas chromatograph matched with Thermo TSQ9000 triple quadrupole mass spectrometry. The instrument was equipped with TG-5MS (30 m × 0.25 mm × 0.25 μm, Thermo, MA, USA) chromatographic column and GHAJ-5190-4048 straight ultra-high inert liner tubes (Agilent, CA, USA). The carrier gas was ultra-high purity helium (≥99.999%) with a constant flow rate of 1 mL·min^−1^ and splitless injection mode. The oven temperature program was customized as follows: initially at 35 °C for 5 min, increased to 75 °C at a ramp of 5 °C·min^−1^ maintained for 3 min, raised to 150 °C at a rate of 3 °C·min^−1^ maintained for 3 min, and eventually increased to 250 °C at a ramp of 12 °C·min^−1^ maintained for 1.5 min. We operated electronic impact (EI) at a voltage of 50 eV with source temperature of 300 °C and transmission line temperature of 280 °C to generate mass spectra. Full scan mode was set with a scanning range of 35–500 *m/z*.

### 2.4 Qualitative and semi-quantitative analysis of VOCs

We used the deconvolution approach to analyze all peaks in OpenChrom 1.5 (Lablicate GmbH, https://www.openchrom.net/) via AMDIS software. VOCs were identified by NIST 2017 library and only VOCs with MS match scores greater than 80% were retained. The peak areas of VOCs were calculated by OpenChrom 1.5. The concentrations of the VOCs in the tomato were measured by the internal standard method and the calculation formula was referenced from Wei et al. (2021). The concentrations were calculated based on the peak area ratio to the internal standard (2-octanol) and were expressed in μg·kg^−1^. These values represented semi-quantitative estimations, not absolute concentrations. We calculated the retention index (RI) of each component using n-alkanes (C8-C40) as described by Wei et al. (2024). To ensure high confidence in compound identification, both RI and MS spectral similarity were considered. VOCs with RI deviations exceeding ±20 units or with low MS similarity (<80%) were not considered reliably identifiable. Eventually, for selected VOCs, ion spectra were manually examined and compared with library entries.

### 2.5 Calculation of relative odor activity values (rOAVs)

rOAVs are commonly employed to evaluate the contribution of aromatic compounds. rOAV was calculated from the ratio of the actual concentration of a certain ingredient to its nasal olfactory threshold (Ma et al., 2025). The VOC concentrations were determined by GC–MS/MS, and their odor thresholds were obtained from literature and listed in Table S3. It was generally considered to have an olfactory characteristic when the rOAV of the VOC was greater than 1 (Ma et al., 2025).

### 2.6 Electronic nose (*E*-nose) analysis

*E*-nose analysis was conducted with slight modifications to the previous methodology (He et al., 2024). Briefly, we ground a 2 g sample into powder using liquid nitrogen and transferred it into a 50 mL brown headspace vial. An amount of 2 mL of saturated CaCl₂ solution and 0.2 mL EDTA/NaOH (pH = 7.5) were added, and the brown headspace vial was tightly sealed with a PTFE‑silicon gasket. Afterward, the sealed vial was sonicated at 50 °C for 40 min. After sonication, the samples were immediately analyzed using the PEN 3.5 *E*-nose (Airsense Analytics GmbH, Germany) equipped with ten metal-oxide sensors. Each sensor corresponded to sensitive VOCs as described by He et al. (2024). The detection parameters were set as follows: injection flow rate was 400 mL·min^−1^, rinse time was 60 s, sensor zero time was 5 s, pre-sampling time was 5 s, measurement time was 180 s. All sensor response data were processed by WinMuster 1.6 (Airsense Analytics GmbH, Germany). Three biological replicates were performed for each tomato variety. The stabilized measurement at 170 s was selected to calculate the average values and plot radar charts.

### 2.7 Machine learning algorithms

Machine learning algorithms, including least absolute shrinkage and selection operator (LASSO), ridge regression, elastic net, light gradient boosting machine (LightGBM), support vector machine (SVM), and random forest, were utilized for classification purposes. Previous reports have indicated that machine learning models constructed with small datasets may lead to several limitations, including model overfitting, significant bias, inadequate data representativeness, and so on (Dou et al., 2023). To mitigate overfitting risks associated with the limited number of tomato varieties, several measures were implemented during dataset preparation and model training. Firstly, as an internal robustness assessment, within-color pairwise ratios were calculated in the GC–MS/MS dataset (for example, B1/B2, B1/B3 to B3/B4; ratios were only computed within the same color group) Importantly, the ratio-transformed data did not increase the number of independent biological observations and were used only as an internal stability check rather than as additional training samples for external validation. We extracted 3 stabilizing values (169–171 s) in the *E*-nose data for each sample. These data were then processed in the same manner as the GC–MS data described above. Secondly, the data was randomly split 70% as the training set and 30% as the test set. Thirdly, we employed 10-fold cross-validation for all model training and parameter tuning, and model performance was evaluated using a test set. Lastly, hyperparameter optimization was conducted using grid search within a resampling framework, which reduced the risk of overfitting. Overall, an internal ratio-based stability assessment, 10-fold cross-validation, an independent test split, and grid-based hyperparameter tuning were jointly applied to reduce overfitting risk during model construction. Receiver operating characteristic (ROC) curves, area under the curve (AUC), accuracy, precision recall, and F1-scores were employed to evaluate the model performance, calculated as described by Gan et al. (2024). All models were developed in RStudio (version 2025.09.1) using the “tidymodels” package in R.

### 2.8 Identification of fruit color-indicating compounds

Partial least squares discriminant analysis (PLS-DA) was performed using SIMCA (14.1, UMETRICS, Umea) software to obtain the variable importance in projection (VIP) scores for each component. The Spearman correlation coefficient was calculated using the MetaboAnalyst online tool (https://www.metaboanalyst.ca). We constructed the random forest model using the MetaboAnalyst, setting 500 decision trees, and obtained the feature importance for each component. Specifically, 26 characteristic compounds were identified by the rOAV analysis; thus, the top 26 compounds based on scores from all methods were chosen for further analysis. The scores of each compound in each method were normalized using Min-Max Scaling in RStudio, and the average values were calculated as the indicative score. For the screened fruit color-indicating compounds, two preliminary criteria were required: an indicative score > 0.1 and scoring by at least two methods. To obtain the final color-indicating compounds, each candidate compound was further subjected to significance testing by Duncan's method (*P* < 0.05). A slightly modified identification method was referenced from (Zhang, Meng, et al., 2021).

### 2.9 Statistical analysis

We adopted RStudio to conduct analysis of variance (ANOVA) and PCA. Significance analysis was conducted by the method of Duncan's (*P* < 0.05). All charts were prepared by RStudio.

## 3 Results

### 3.1 Qualitative and semi-quantitative analysis of volatile compounds (VOCs) from 16 tomato varieties

**Fig. 2:**
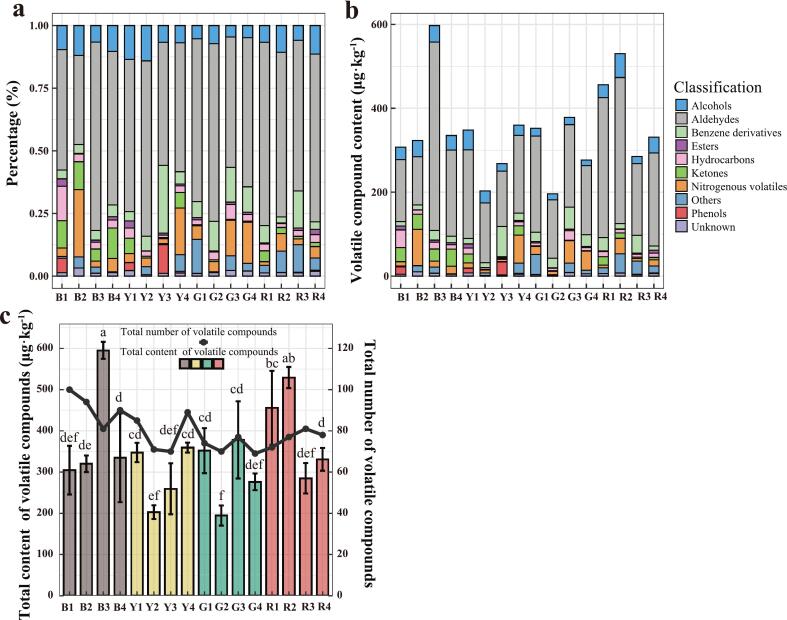
(a) The percentage of different types of VOCs in different tomato varieties. (b) The contents of different types of VOCs. Each value denoted the average of 3 biological replicates. (c) The total contents and quantities of VOCs in different tomato varieties. Each value represented the mean ± standard deviation of 3 biological replicates. Bars meant by the same letter showed no significant difference at *P* < 0.05 based on Duncan's test.

A total of 154 volatile compounds (VOCs) were detected from the flesh of 16 tomato varieties using HS-SPME-GC–MS/MS, which were classified into 10 categories, including 21 alcohols, 29 aldehydes, 17 benzene derivatives, 8 esters, 22 hydrocarbons, 10 ketones, 4 nitrogen-containing volatiles, 6 phenols, 5 others, and 32 unknowns, with total contents in the varieties ranged from 194.56 μg·kg^−1^–595.27 μg·kg^−1^ (Table S2). The total number of volatiles in the Y varieties was the highest, specifically 126; however, 93 volatiles were found among all G varieties, with the lowest number found among blocks of varieties. In addition, the total ion chromatograms (TICs) of VOCs from the flesh of 16 tomato varieties were shown in Fig. S2, implying differences in the composition of VOCs in tomatoes. Among all varieties, the aldehyde contents were highest in the brown tomato variety B3, with 448.93 μg·kg^−1^, accounting for approximately 75.42% (Fig. 2a, b). As shown in Fig. 2c, B3 also had the highest total VOC content (595.27 μg·kg^−1^) among all varieties, comprising 81 VOCs. Notably, B3 was not the variety with the greatest number of VOCs; variety B1 contained 100 VOCs. It is worth noting that there were no statistically significant differences with the R2 variety compared to B3 variety. In contrast, the lowest total VOC content in green tomato variety G2 was 194.56 μg·kg^−1^ and the number of VOCs was 70. The results indicated that the composition of VOCs in the flesh of tomato varieties was diverse, possibly resulting in differences in flavor. Among the 154 VOCs, 32 were categorized as unknown. The peaks of 32 VOCs showed clear and repeatable signals but lacked reliable spectral matches in the NIST library. We used the retention index for identification, but the results were mismatched. In future studies, we plan to apply higher-resolution MS techniques and authentic standards to further identify these unknown compounds.

### 3.2 Relative odor activity values (rOAVs) analysis

**Fig. 3:**
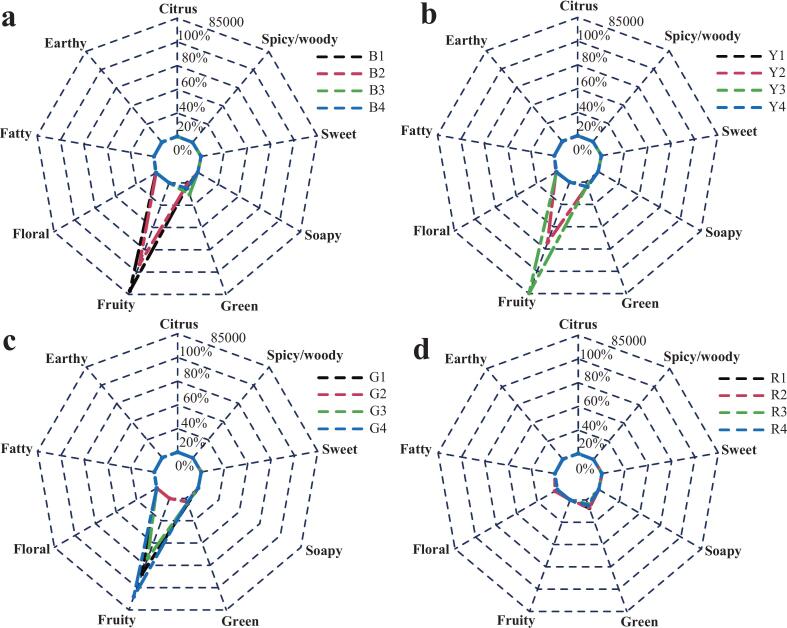
Odor fingerprints of (a) brown, (b) yellow, (c) green, and (d) red tomato varieties based on rOAV analysis. Each value indicated the average of 3 biological replicates. (For interpretation of the references to color in this figure legend, the reader is referred to the web version of this article.)

A total of 26 potential VOCs were screened to be considered as characteristic aroma components (rOAV >1) of tomato flesh in this study (Table S3). The number of aldehydes (11 species) was dominant among 26 potential VOCs, and the rOAV of hexanal exceeded 2000 in all varieties. Notably, damascenone showed the highest rOAV (30,775.69–84,690.21), which largely reflected its extremely low odor threshold reported in the literature (Table S3). However, given threshold variability and matrix effects in complex food systems, this value should be interpreted cautiously and mainly as a prioritization indicator. As previously reported, odor characteristics of 26 potential VOCs were divided into 9 categories, including fruity, green, sweet, floral, citrusy, earthy, spicy/woody, fatty, and soapy (Wei et al., 2024; Zhang et al., 2023). These 9 odor characteristics systematically described the overall aroma profile of tomato varieties with four fruit colors (Fig. 3). Fruity aroma dominated in some of the brown, yellow, and green tomato varieties, whereas the green note was relatively more abundant in the red varieties (Fig. 3). The absence of damascenone in the tested red tomato varieties might contribute to their weaker fruity aroma, although its perceptual impact requires sensory validation. Additionally, hexanal contributed significantly to the green aroma, with its rOAV distribution ranging from 2,138.51 to 8,772.87 in all tomato varieties (Fig. 3; Table S3). Furthermore, the earthy smell was mainly attributed to two VOCs: 1-octen-3-one and 2-isobutylthiazole. Interestingly, 1-octen-3-one was detected in all the tested tomato varieties, whereas 2-isobutylthiazole was only determined in some varieties. The above results demonstrated that the differential rOAV distributions of characteristic VOCs might contribute to distinct flavor profiles in tomato varieties with different fruit colors.

### 3.3 *E*-nose patterns and correlation with volatile compounds

**Fig. 4:**
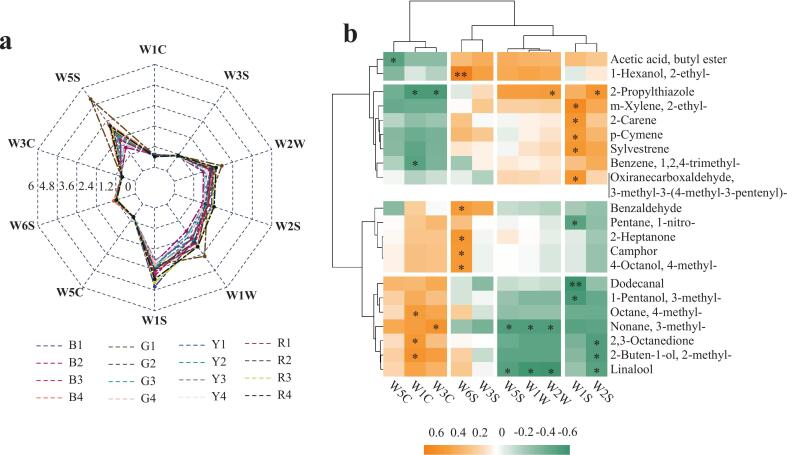
*E*-nose response patterns and their correlations with GC–MS/MS volatiles in tomato flesh. (a) The aroma profiles of 16 tomato varieties constructed by *E*-nose. (b) The correlation plot of 10 sensors and VOCs from GC–MS/MS data. Correlation analysis was conducted by the Spearman algorithm. “*” showed significant correlation (*P* < 0.05). “**” showed very significant correlation (*P* < 0.01).

As shown in Fig. 4a, 5 *E*-nose sensors (W5S, W1S, W1W, W2W, W2S) had a high response and a wide range of response values, illustrating that the flesh of the tested tomato varieties was rich in nitrogen oxides, methyl compounds, organosulfides, terpenes, and oxygen-containing VOCs.

**Fig. 5:**
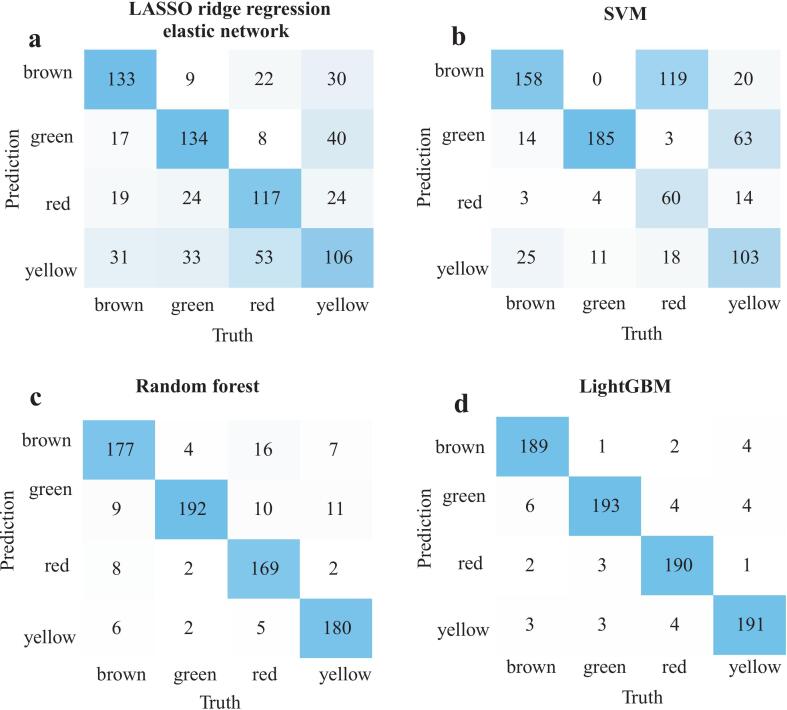
Confusion matrices were output by (a) LASSO ridge regression elastic network, (b) SVM, (c) random forest, and (d) LightGBM algorithms using *E*-nose data.

Correlation analysis between the 10 sensors and all identified VOCs (excluding unknowns) was performed according to the method of Wei et al. (2024). Only compounds that showed significant correlations with the 10 sensors were retained. The *E*-nose sensors were clustered into four categories: W5S, W1W, and W2W sensors were classified into a group, and W1S and W2S sensors were grouped into a category (Fig. 4b). Notably, 1-hexanol, 2-ethyl-, described as fruity and sweet odors, demonstrated a highly significant positive correlation with the W6S sensor (*P* < 0.01), suggesting strong sensor responsiveness to branched-chain alcohols. In contrast, a significant negative correlation (*P* < 0.01) was observed between sensor W1S and dodecanal (fatty, violet, soapy smells), a medium-chain aliphatic aldehyde, indicating possible sensor suppression or limited sensitivity to less volatile, long-chain aldehydes. These findings highlighted the differential selectivity of *E*-nose sensors and their potential in detecting class-specific volatile compounds.

### 3.4 Classification and prediction of tomato fruit colors using *E*-nose data and machine learning

To evaluate whether the *E*-nose could effectively differentiate tomato varieties by fruit color, we applied several machine learning algorithms to classify samples using *E*-nose data. As shown in Table 1, all models achieved moderate to high accuracy using the original *E*-nose data, with LightGBM ranking highest, followed by SVM and random forest. These results indicated that the *E*-nose sensor responses contained sufficient information to distinguish tomato color, particularly when nonlinear models were used. As an internal stability assessment, the same algorithms were additionally applied to a within-color pairwise ratio transformation of the *E*-nose data, and the corresponding confusion matrices were shown in Fig. 5. Model accuracy slightly decreased, but AUC remained above 0.84, indicating that the relative patterns captured by the ratio transformation yielded consistent results across models. Notably, all models maintained the same ranking trends (LightGBM > random forest > SVM > LASSO ridge regression elastic network), reflecting robustness across algorithms. Overall, the *E*-nose combined with LightGBM showed the highest performance within our dataset, and the ratio transformation served as an internal check on the stability of the observed color-related patterns.

**Table 1:** Comparison of model performance between original data and pairwise ratio data by *E*-nose.

Dataset type	Machine learning algorithm	Accuracy	Precision	Recall	F1-scores	AUC
Original data	LASSO ridge regression elastic network	0.76	0.54	0.55	0.52	0.81
	LightGBM	1.00	1.00	1.00	1.00	1.00
	SVM	0.75	0.78	0.75	0.76	0.94
	Random forest	0.66	0.68	0.66	0.76	0.84
Pairwise ratio data	LASSO ridge regression elastic network	0.61	0.62	0.61	0.61	0.84
	LightGBM	0.95	0.95	0.95	0.95	1.00
	SVM	0.63	0.66	0.63	0.61	0.93
	Random forest	0.90	0.900	0.90	0.90	0.99

**Note:** The values were rounded to two decimal places.

### 3.5 Classification and prediction of tomato fruit colors using GC–MS/MS data and machine learning

**Fig. 6:**
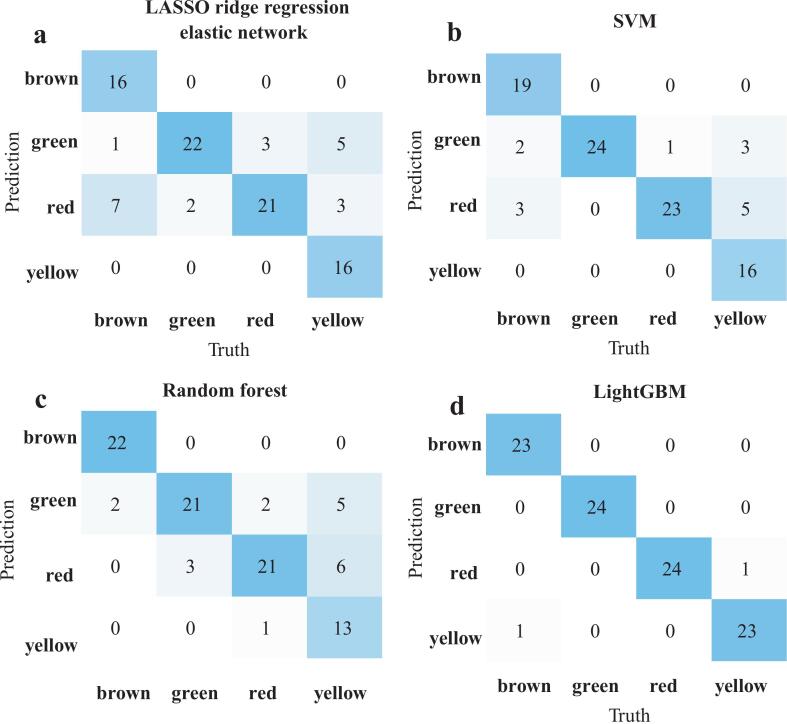
Confusion matrices were output by (a) LASSO ridge regression elastic network, (b) SVM, (c) random forest, and (d) LightGBM algorithms using GC–MS/MS data.

We adopted 4 machine learning classification algorithms to evaluate the effectiveness in classifying the fruit color of tomato using GC–MS/MS data. As shown in Table 2, all models trained on the original dataset achieved high classification performance, with LightGBM showing the highest performance in the current dataset (accuracy = 1.00, AUC = 1.00). Using the pairwise-ratio dataset resulted in a modest decline compared to the original data, but all models retained high accuracy (LightGBM = 0.98, SVM = 0.85, Random Forest = 0.80, LASSO = 0.78) and AUC values above 0.92. Importantly, the models maintained the same performance ranking (LightGBM > SVM > random forest > LASSO ridge regression elastic network), indicating that the ratio transformation preserved the essential discriminative information. The ROC curves for all the models were basically clustered near the top-left corner (Fig. S3). The confusion matrices for all models suggested that 4 classification algorithms had misclassified the fruit color of tomato, with the LightGBM having the fewest misclassifications, where brown was misclassified as red (Fig. 6). Taken together, these results suggested that GC–MS/MS-derived VOC features might enable within-dataset discrimination of fruit color in the tested varieties, while the pairwise-ratio transformation should be regarded as an internal stability check rather than evidence of model generalizability.

**Table 2:** Comparison of model performance between original data and pairwise ratio data byGC–MS/MS.

Dataset type	Machine learning algorithm	Accuracy	Precision	Recall	F1-scores	AUC
Original data	LASSO ridge regression elastic network	1.00	0.95	0.94	0.94	1.00
	LightGBM	1.00	1.00	1.00	1.00	1.00
	SVM	1.00	0.90	0.88	0.87	0.98
	Random forest	1.00	1.00	1.00	1.00	1.00
Pairwise ratio data	LASSO ridge regression elastic network	0.78	0.84	0.78	0.78	0.92
	LightGBM	0.98	0.98	0.98	0.98	1.00
	SVM	0.85	0.89	0.85	0.85	0.95
	Random forest	0.80	0.83	0.80	0.80	0.97

**Note:** The values were rounded to two decimal places.

### 3.6 Identification of fruit color-indicative candidates by multivariate analysis

**Fig. 7:**
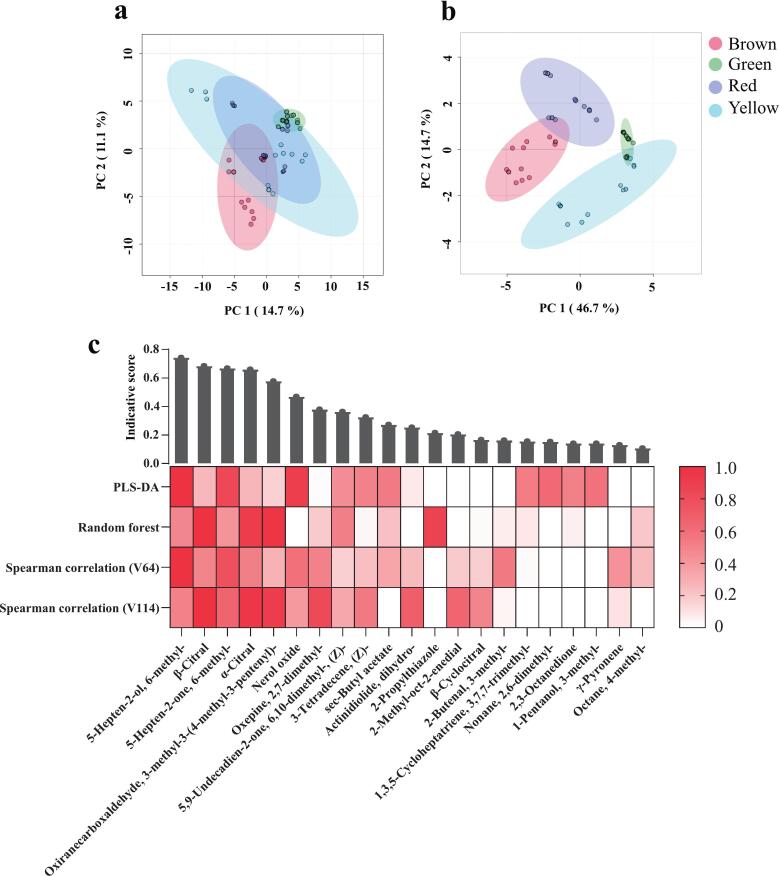
Screening of potential fruit color-indicating VOCs and PCA visualization. (a) PCA result for all GC–MS/MS data. (b) PCA result for 18 fruit color-markers. (c) Color indicative VOCs were screened by PLS-DA, random forest, and Spearman's correlation.

The above analyses indicated that machine learning algorithms using GC–MS/MS-derived VOC features might effectively categorize tomato varieties by fruit color. Therefore, we employed a modified approach based on Zhang, Meng, et al. (2021) to screen for fruit color-indicative VOCs. PLS-DA analysis identified 41 VOCs with VIP values greater than 1. Twenty-six characteristic aroma volatiles of tomato flesh were selected by previous rOAV analysis; thus, the top 26 compounds based on VIP values were chosen for further analysis. Similarly, the top 26 VOCs were identified based on variable importance in the random forest analysis. Two volatiles, 5-hepten-2-ol, 6-methyl- and β-citral, were ranked first (scores of 2.57 and 0.047, respectively) in terms of VIP and variable importance values. Therefore, the top 26 volatiles at two levels were selected for the Spearman correlation analysis. The average of the indicative score for each VOC was calculated using the min-max normalization from the four evaluation scores (Fig. 7c). A total of 18 VOCs were preliminarily identified as fruit color-indicative candidates, with 5-hepten-2-ol, 6-methyl- having the highest mean scores. To explore the discriminative power of these 18 fruit color-indicative candidates, PCA was conducted on samples from tomato varieties of different colors. As shown in Fig. 7a, PCA using all VOCs explained 25.8% of the variance (PC1 + PC2) and showed poor separation of the tomato varieties by fruit color. In contrast, using 18 fruit color-indicative candidates significantly improved the separation of varieties by fruit color in the PCA results (Fig. 7b). PC1 and PC2 together explained 61.4% of the total variance, indicating a high contribution. The above results indicated that these 18 fruit color-indicative candidates might effectively distinguish fruit colors in the tested tomato varieties.

### 3.7 Analysis of potential fruit color-indicative compounds in differentially colored tomato varieties

**Fig. 8:**
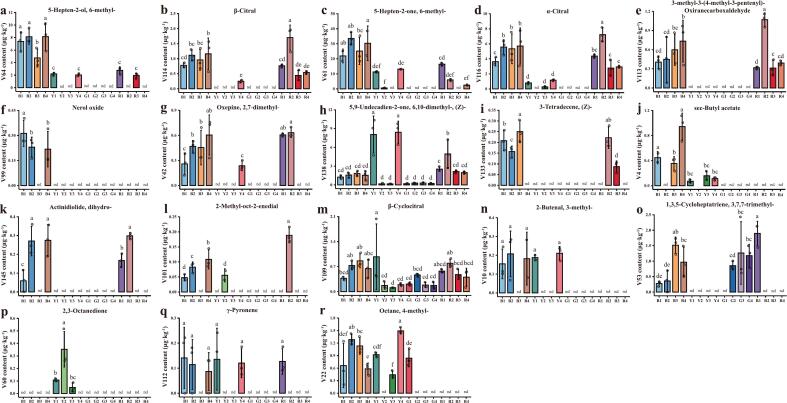
(a-r) Contents of potential 18 fruit color-indicating VOCs of 16 tomato varieties. Each value showed the mean ± standard deviation of 3 biological replicates. Bars meant by the same letter represented no significant difference at *P* < 0.05 based on Duncan's test.

The content of 5-hepten-2-ol, 6-methyl- in brown tomato was significantly higher than that of other color, suggesting that it can be used as a marker for the identification of brown tomato (Fig. 8a). Nerol oxide was detected only in brown tomato (Fig. 8f). Similarly, 2,3-octanedione was specifically present in yellow tomato (Fig. 8p). High levels of 5,9-undecadien-2-one, 6,10-dimethyl-, (*Z*)- were found in the yellow Y1 and Y4 varieties (Fig. 8h). Some compounds were specific to either the brown/red or yellow/green groups, including α-citral (Fig. 8d), 3-methyl-3-(4-methyl-3-pentenyl)-oxiranecarboxaldehyde (Fig. 8e), 3-tetradecene, (Z)- (Fig. 8i), and actinidiolide, dihydro- (Fig. 8k). Interestingly, our results implied that green tomatoes lacked many color-indicating indicative compounds, such as, 5-hepten-2-ol, 6-methyl-, β-citral, 5-hepten-2-one, 6-methyl-, α-citral, and others. Furthermore, β-cyclocitral and 5,9-undecadien-2-one, 6,10-dimethyl-, (Z)- were not specific to any fruit color group (Fig. 8h, m) and were therefore not considered as color-indicating compounds. Ultimately, 16 compounds were selected as fruit color-indicative markers. These results indicated that certain compounds were present only in specific fruit-color groups of the tested varieties, further illustrating that they might be useful markers for distinguishing the fruit color. Meanwhile, the findings suggested that these fruit color-indicating compounds might play an important role in the formation of the unique flavor profiles associated with different tomato fruit colors.

## 4 Discussion

Although VOCs account for a small proportion of the fresh weight of fruits and vegetables, their combination at a certain level can produce distinctive and characteristic flavors (Wei et al., 2024). In this study, a total of 154 VOCs were identified in the flesh of 16 tested tomato varieties using HS-SPME-GC–MS/MS (Table S2). Among them, aldehydes (29 species), hydrocarbons (22 species), and alcohols (21 species) were the most abundant. Li et al. (2022) showed that aldehydes contributed to the green odor and enhanced the freshness of tomatoes, while alcohols impart sweetness and play a key role in flavor enhancement. Hydrocarbons helped balance and intensify the overall flavor profile (Li et al., 2022). These findings suggested that the tested tomato varieties might primarily exhibit green and sweet aroma characteristics. Additionally, the total VOC contents in the tested tomato varieties ranged from 194.56 μg·kg^−1^ to 595.27 μg·kg^−1^, which were lower than those in previous studies (Bauchet et al., 2017; Cheng et al., 2020). Firstly, the results might be attributed to differences in tomato varieties and the climatic conditions of the cultivation regions compared to previous studies. Meanwhile, this study was conducted using samples grown in a single location and season to ensure uniform conditions, which might limit the generalizability of the results. Therefore, future studies should include multi-location or multi-season trials to validate the robustness of the findings. Secondly, in this study, 2-octanol was used as a single internal standard for semi-quantitative analysis of all VOCs. We acknowledge that using a single internal standard for chemically diverse compounds might introduce bias, especially considering differences in volatility and polarity, as well as variations in ion fragment signal intensity or peak area among different compounds. Future work will consider compound-specific calibration and matrix-matched standards to improve quantitative accuracy. Thirdly, in terms of technology might have contributed to the relatively low total VOC concentrations observed in this study. Matrix effects, such as interactions between volatiles and liquid, might have reduced volatile release. Variations in SPME fiber properties, incubation conditions, or desorption settings could affect compound recovery, resulting in differences in extraction efficiency. Instrumental factors, for example, detector sensitivity and scan parameters, might influence detection limits. These technical aspects should be considered, and future work could optimize or develop analytical procedures to improve recovery and comparability. It was noteworthy that the use of flesh in this study might lead to a reduction in total VOC contents and changes in aroma profile. Previous reports noted that the pericarp of tomato exhibited the highest total VOC content; locular gel and seed had the most abundant aldehydes; and the stem end tissue lacked the aroma profiles of tomato fruits (Li, Di & Bai, 2019). We speculated that the low total VOC levels might be due to the seed removal or the specific growing conditions. Interestingly, Guan et al. (2025) cut tomatoes into small pieces to identify 585 VOCs from 7 cherry tomato varieties by GC–MS. By comparison, only 154 VOCs were detected using 16 tomato varieties. Therefore, future studies should compare the effects of different sample preparation methods on tomato aroma patterns. There are several methods, including freeze drying(Zhang, Liu, et al., 2024), juice extraction(Li, Fu, Bao, Li & Zuo, 2019), and grinding into pulp (Zhang et al., 2023), for preparing tomato samples to analyze aroma, but no comparative studies have been conducted. Meanwhile, flavor analysis of whole fruit and different tissues (pericarp, locular gel, seeds) is also worth further exploration.

To date, more than 400 volatile compounds have been detected in tomato fruits, and only approximately 20 VOCs contribute to the characteristic aroma profile (Kelebek et al., 2018). Our results demonstrated that 26 VOCs exhibited rOAVs exceeding 1.0, indicating that these 26 VOCs might affect the characteristic aroma of the tested tomato varieties (Table S3). Similarly to the present study, Cheng et al. (2020) found a total of 22 volatiles with rOAV>1 from 71 tomato varieties, suggesting that these 22 VOCs had a significant effect on the aroma of 71 tomato varieties. In our study, the flesh of tomato varieties was dominated by fruity smell, except in red tomato varieties (Fig. 3). Zhang et al. (2023) similarly implied that fruity and sweet smells contributed unique flavor in tasty tomato. Meanwhile, green, floral, and woody aromas were dominant in the cherry tomato. Interestingly, in another report, fatty and green flavors were significant flavor profiles in cherry tomato (Guan et al., 2024). Moreover, compared to the other fruit-colored tomatoes, the tested red tomato varieties exhibited low intensity of fruity aroma, which might be due to the non-detection of damascenone. The green aroma was the secondary predominant characteristic in the tested tomato varieties. Previous investigation demonstrated that damascenone and hexanal synergistically contribute to the development of complex aroma profiles in tomato fruits (Wang et al., 2016). Overall, the aroma fingerprints of tomatoes with different fruit colors show distinct differences. However, the underlying mechanisms remain unclear, particularly whether the biosynthetic pathway of damascenone interacts with pigment pathways. Although this study identified key aroma compounds based on rOAV and multivariate analysis, it did not include sensory validation by trained panel tests or consumer evaluations. As flavor is a complex trait shaped by both volatile and non-volatile factors, the lack of direct sensory or taste measurements (such as sweetness, acidity, liking scores) limits conclusions about the functional impact of these compounds. Therefore, future studies should integrate aroma profiles with physicochemical attributes and sensory assessments to better understand how specific volatiles contribute to perceived flavor and consumer preference. Additionally, we noted that the rOAV of damascenone exceeding 30,000 was an extreme value. In this study, rOAV values were calculated using odor thresholds in water compiled from the literature (Table S3), which were typically determined in simplified matrices (such as water and air) and might not be directly transferable to the tomato matrix (Perry & Hayes, 2016). Future work should employ GC-olfactometry (GC-O) and omission/recombination to confirm the sensory relevance of these VOCs.

The *E*-nose system can quickly develop the aroma profile of a sample and has been widely adopted for identifying the varieties and production areas of horticultural plants(Zhang, Xue, et al., 2024). It was noteworthy that the *E*-nose results were not fully supported by the GC–MS/MS data in our results. While the *E*-nose showed lower responses for aldehyde-, hydrocarbon-, and alcohol-sensitive sensors, the GC–MS/MS analysis detected a wide range of aldehyde, hydrocarbon, and alcohol compounds. The inconsistency between *E*-nose and GC–MS/MS results might be partly due to differences in sample preparation procedures. Firstly, fewer samples and agents were used in *E*-nose analysis compared to HS-SPME sample preparation. Furthermore, the *E*-nose sample was sonicated at 50 °C for 40 min, whereas the HS-SPME sample was just equilibrated and extracted at 50 °C. Interestingly, Wei et al. (2024) utilized the same method to prepare samples for GC–MS and *E*-nose and found that hydrocarbons, aldehydes, and esters were abundant in GC–MS data, but aromatic components, sulfur compounds, and nitrogen oxides were abundant in *E*-nose data. The result indicates that even when using the same preparation method, variations can still occur. Therefore, we hypothesized that this discrepancy was caused by the utilization of extracted fiber by HS-SPME and the non-utilization of the *E*-nose. In addition, the principles of the two instruments were not the same, resulting in the independence of the results. Specifically, GC–MS/MS physically separates each VOC by chromatography and then identifies and quantifies them via mass spectrometry (Patil et al., 2023; Wei et al., 2023). This method provides high-resolution qualitative and quantitative data. However, GC–MS/MS focuses solely on the chemical identity and abundance of each compound, without considering potential interactions among VOC that may influence sensory perception. By contrast, the *E*-nose simulates human olfactory function through a non-specific sensor array that detects the overall headspace profile (Wei et al., 2023). This equipment can generate a composite signal pattern based on the collective response of sensors to different chemical classes, but cannot identify individual compounds. Hence, the different operating mechanisms of the two instruments might explain the inconsistency between the *E*-nose and GC–MS/MS findings.

In recent years, machine learning has been applied to distinguish the origin, type, and quality of food, as well as to predict and evaluate storage time (Ji et al., 2023). To model the relationship between aroma profiles and tomato color groups, we employed 4 machine learning algorithms, including LASSO ridge regression elastic net, random forest, SVM, and LightGBM. Due to their wide application in metabolomics and food flavor studies and suitability for small sample sizes, these models were selected in this study. For example, LASSO was applied in baijiu aging research to screen key aging-related volatiles and achieved better model interpretability in predicting sensory stages (An et al., 2024). Faquih et al. (2025) employed ridge regression study to construct a robust age prediction model using >100 metabolite variables in human metabolomics. Elastic net, which balances the sparsity of LASSO and the stability of ridge regression, was effectively built in a flavor study of pepino (*Solanum muricatum*) (Sun et al., 2022). In terms of nonlinear models, random forest was successfully used in classifying plant species in herbal mixtures based on pseudotargeted metabolomic data (Cai et al., 2023). Their results indicated that the random forest model achieved >95% accuracy and provided robust variable importance scores. Similarly, (Li, Han, Mei, Wang & Han, 2024) constructed SVM to predict the storage time of organic green tea samples based on VOC profiles. LightGBM, a boosting-based model optimized for speed and performance, was utilized to predict association disease with metabolite(Zhang, Lei & Liu, 2021). Within the current dataset, LightGBM yielded the highest discrimination performance for both *E*-nose and GC–MS/MS data in our study. The ratio transformation produced broadly consistent trends and was therefore used only as an internal stability check, while external datasets are required for further validation. Majchrzak et al. (2018) employed GC–MS data of red and white wines to develop an SVM classification model. The results showed that the SVM model exhibited high accuracy in distinguishing between white and red wines. Furthermore, the authors observed that the SVM model could classify wine samples based on vineyard geographical region, yeast type, post-fermentation treatment, and fermentation temperature (Majchrzak et al., 2018), indicating that the SVM model can also be applied to wine adulteration detection. Feng et al. (2018) constructed SVM and partial least squares regression (PLSR) models to predict the postharvest quality of cucumbers, using nuclear magnetic resonance (the state and migration of water molecules), *E*-nose data as inputs, and cucumber firmness, pH, and storage time as outputs. They found that the SVM model based on *E*-nose data exhibited optimal predictive performance for postharvest quality indices, indicating a close association between quality changes during cucumber storage and flavor characteristics. However, the correlation with water status and migration was relatively low (Feng et al., 2018). Our results provided insights for applications in the development of *E*-nose, GC–MS/MS, and related algorithms. Although the pairwise ratio dataset provided stable classification results, several limitations should be noted. Because the ratios were calculated from the same samples, the number of independent observations did not increase, which might limit model generalization. Meanwhile, ratio calculation could amplify noise when low-abundance compounds were involved and might weaken absolute concentration information. Additionally, the model constructed in this study belonged to an in-data task. Thus, our further work will focus on external datasets to verify the performance of the models.

It has been shown that the composition of VOCs differs among various types of tomatoes. For instance, the aroma profiles of tasty tomatoes, regular tomatoes, and cherry tomatoes exhibited significant differences (Zhang et al., 2023). Additionally, the authors found that 1-octene-3-one contributed a mushroom-like aroma and was most abundant in cherry tomato varieties. (Zhang, Ye, et al., 2024) examined VOCs in 4 accessions from 3 tomato subgroups to find that pentanol was identified only in green tomato fruits and 1-pentanol in red tomato fruit, suggesting fruit colors might affect the aroma composition in tomatoes. In a recent report, citronellyl isobutyrate was identified exclusively in yellow tomato cultivars, which also showed the lowest overall volatile content compared to pink, brown, and red tomato cultivars (Guan et al., 2025). In this study, we observed that 16 compounds were detected uniquely in tomatoes of specific colors, which was inconsistent with previous findings. For example, we detected nerol oxide exclusively in brown tomato varieties (Fig. 8f) and 2,3-octanedione exclusively in yellow tomato varieties (Fig. 8p). Moreover, the 16 color-indicative compounds originated from multiple biosynthetic pathways. A substantial number are derived from carotenoid degradation, including 5-hepten-2-ol, 6-methyl- (Gao et al., 2008), β-citral (Ilg et al., 2014), 5-hepten-2-one, 6-methyl- (Gao et al., 2008), α-citral (Lewinsohn et al., 2005), 3-methyl-3-(4-methyl-3-pentenyl)-oxiranecarboxaldehyde (Lewinsohn et al., 2005), actinidiolide, dihydro- (Lewinsohn et al., 2005), 2-methyl-oct-2-enedial (Ilg et al., 2014). Previous studies have shown that dark-colored tomatoes were rich in carotenoids, suggesting that dark-colored tomatoes contained high levels of volatiles produced by carotenoid degradation (Lewinsohn et al., 2005). In our study, the 7 color markers driven by carotenoid degradation exhibited high levels in partially brown and red tomatoes (Fig. 8). 2,3-Octanedione, derived from fatty acid oxidation in microalgae (Kumari, Cna'ani, Didi-Cohen, Tzin & Khozin-Goldberg, 2020), was only detected in yellow tomatoes in our study (Fig. 8p). This compound might reflect early-stage lipid peroxidation before the full onset of carotenoid accumulation. In contrast, nerol oxide, a derivative of monoterpene biosynthesis (Wust et al., 1999), was specific to brown tomatoes (Fig. 8f), possibly reflecting cultivar-specific terpene activity or genetic background. Besides, several color markers detected in this study lack clearly defined biosynthetic pathways. These compounds might arise from minor oxidative reactions, cultivar-specific metabolism, or background chemical processes, and their biological or sensory significance remains to be further clarified. Additionally, it is noteworthy that this study utilized only 16 tomato varieties, which might reduce the generalizability of the results. A small sample size might increase the risk of overfitting in statistical models and might not fully capture the variability across broader tomato populations. Future studies with more cultivars, such as wild species and germplasm resources, as well as growing conditions, would help validate and extend these findings.

## 5 Conclusion

In this study, 154 VOCs were analyzed in tomato flesh from 16 tomato varieties using HS-SPM*E*-GC–MS/MS, with aldehydes comprising the largest group. The rOAV analysis identified 26 key aroma compounds that might collectively contribute to flavor characteristics such as fruity, grassy, floral, and earthy notes. The absence of damascenone in red tested tomato varieties might result in their relatively weaker fruity aroma. The synthetic pathways of these 26 key compounds should be focused on in the future. Moreover, the *E*-nose and GC–MS/MS data in conjunction with LightGBM might enable within-dataset discrimination of tomato fruit color in the tested varieties. Future work should involve more tomato varieties and sensory data to improve model robustness and application. In conclusion, our study offered new insights into the instrumental evaluation of volatile profiles associated with fruit color and provided a reference for subsequent sensory validation. Future research will focus on integrating sensory evaluation with instrumental and computational analyses to validate the sensory perception of key aroma compounds.

## CRediT authorship contribution statement

**Junrong Xu:** Writing – review & editing, Writing – original draft, Software, Methodology, Investigation, Formal analysis, Data curation. **Yushi Lu:** Methodology, Investigation. **Jing Cui:** Writing – review & editing, Writing – original draft, Methodology. **Yunzhi Liu:** Methodology, Investigation. **Wenjin Yu:** Project administration. **Changxia Li:** Writing – review & editing, Writing – original draft, Supervision, Project administration, Conceptualization.

## Declaration of competing interest

The authors declare that they have no known competing financial interests or personal relationships that could have appeared to influence the work reported in this paper.

## Data Availability

Data will be made available on request.

## Appendix A Supplementary data

Supplementary material 1 **Supplementary Table S1** List of Tomato varieties tested. **Supplementary Table S2** The composition and content of volatile compounds in 16 tomato varieties by HS-SPME-GC–MS/MS. **Supplementary Table S3 Relative** odor activity values (rOAVs) of the 26 most potent volatile compounds in 16 tomato varieties. ROC curves of (a) LASSO ridge regression elastic network, (b) SVM, (c) random forest, and (d) LightGBM algorithms. Image 1

Supplementary material 2 **Supplementary Fig. S1** The color difference values of fruit from 16 tomato varieties. Note: The values of (a) lightness (L*), (b) greenness/redness (a*), (c) blueness/yellowness (b*), and (d) hue angle (H°) from 16 tomato varieties. Each value showed the mean ± standard deviation of 3 biological replicates. Bars meant by the same letter represented no significant difference at *P* < 0.05 based on Duncan's test. **Supplementary Fig. S2** Total ion chromatogram (TIC) of volatile compounds from 16 tomato varieties. **Supplementary Fig. S3** ROC curves of 4 machine learning classification algorithms. Image 2
