# Supplementary material for: Unraveling the difference in aroma characteristics of tomato flesh with different colors using HS-SPME-GC–MS/MS and E-nose combined with multivariate data analysis
Source: Food Chem X. 2026 Jan 30;34:103594. doi: 10.1016/j.fochx.2026.103594 (PMC12906072; doi:10.1016/j.fochx.2026.103594)
Supplement: Supplementary file 1 — Supplementary material 1 Supplementary Table S1 List of Tomato varieties tested. Supplementary Table S2 The composition and content of volatile compounds in 16 tomato varieties by HS-SPME-GC–MS/MS. Supplementary Table S3 Relative odor activity values (rOAVs) of the 26 most potent volatile compounds in 16 tomato varieties. ROC curves of (a) LASSO ridge regression elastic network, (b) SVM, (c) random forest, and (d) LightGBM algorithms. [file mmc1.docx]

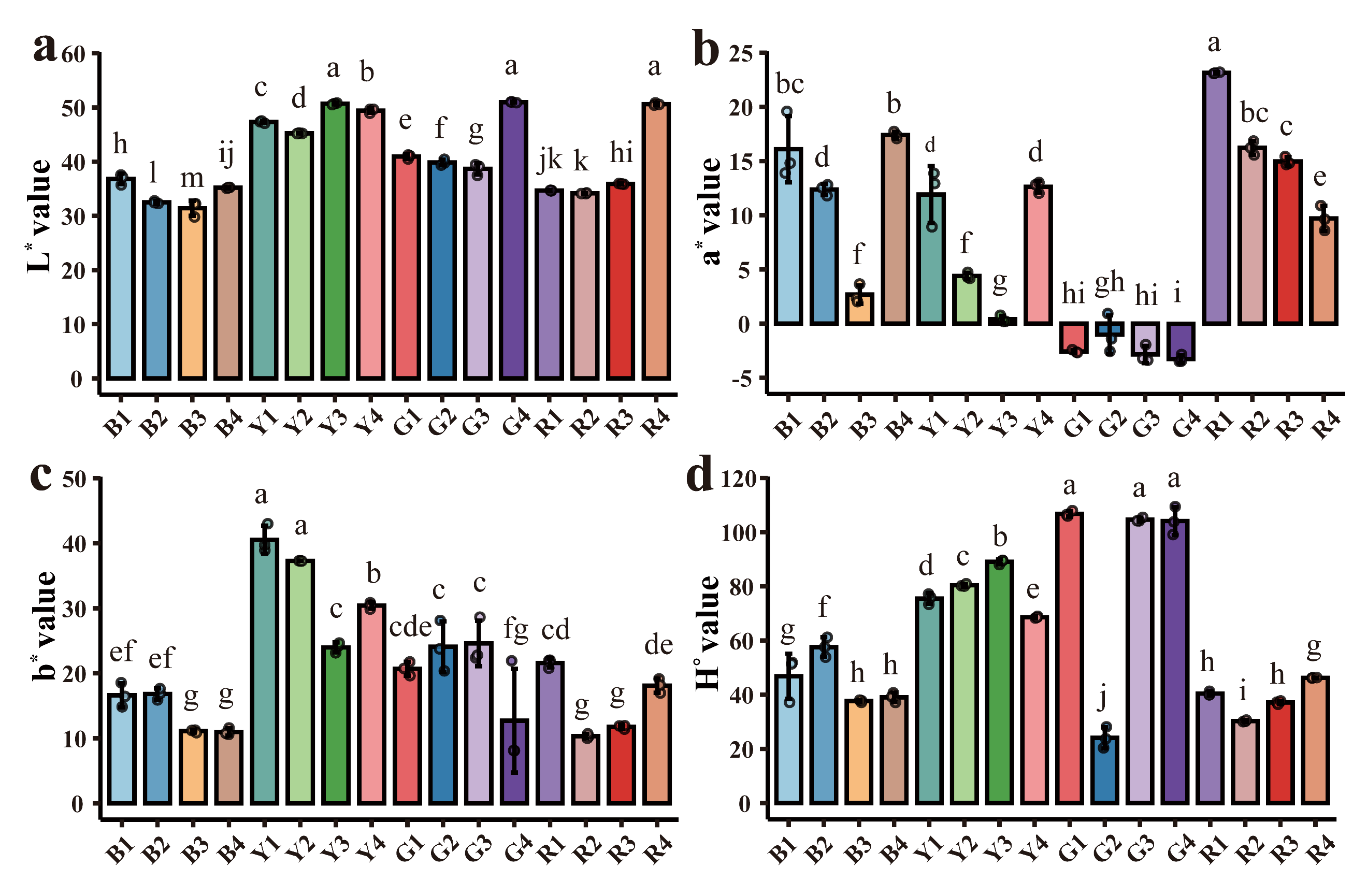


**Fig. S1 The color difference values of fruit from 16 tomato varieties.** The values of (a) lightness (L*), (b) greenness/redness (a*), (c) blueness/yellowness (b*), and (d) hue angle (H°) from 16 tomato varieties. Each value showed the mean ± standard deviation of 3 biological replicates. Bars meant by the same letter represented no significant difference at *P* < 0.05 based on Duncan’s test.

**
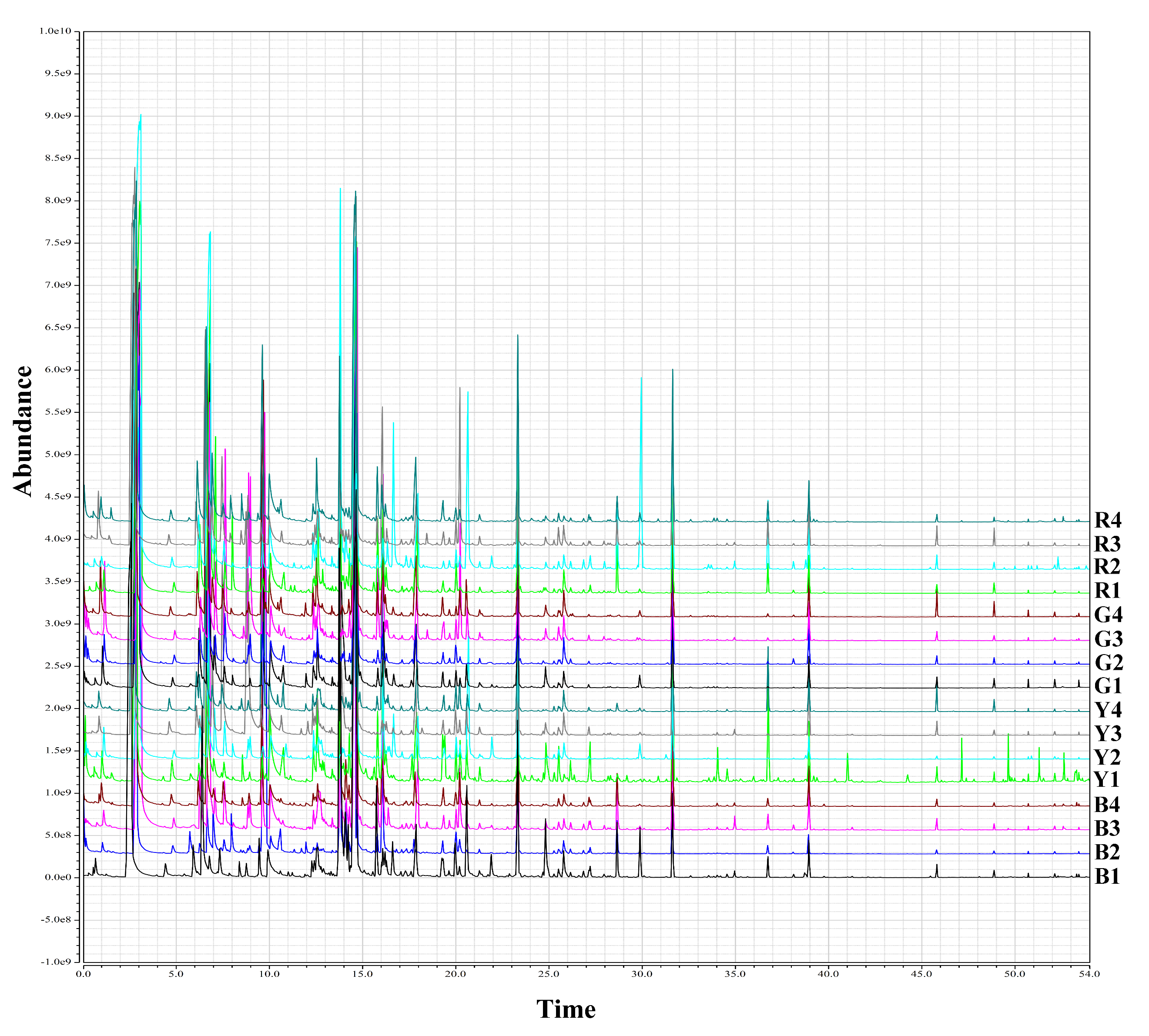
**

**Fig. S2 Total ion chromatogram (TIC) of volatile compounds from 16 tomato varieties.**

**
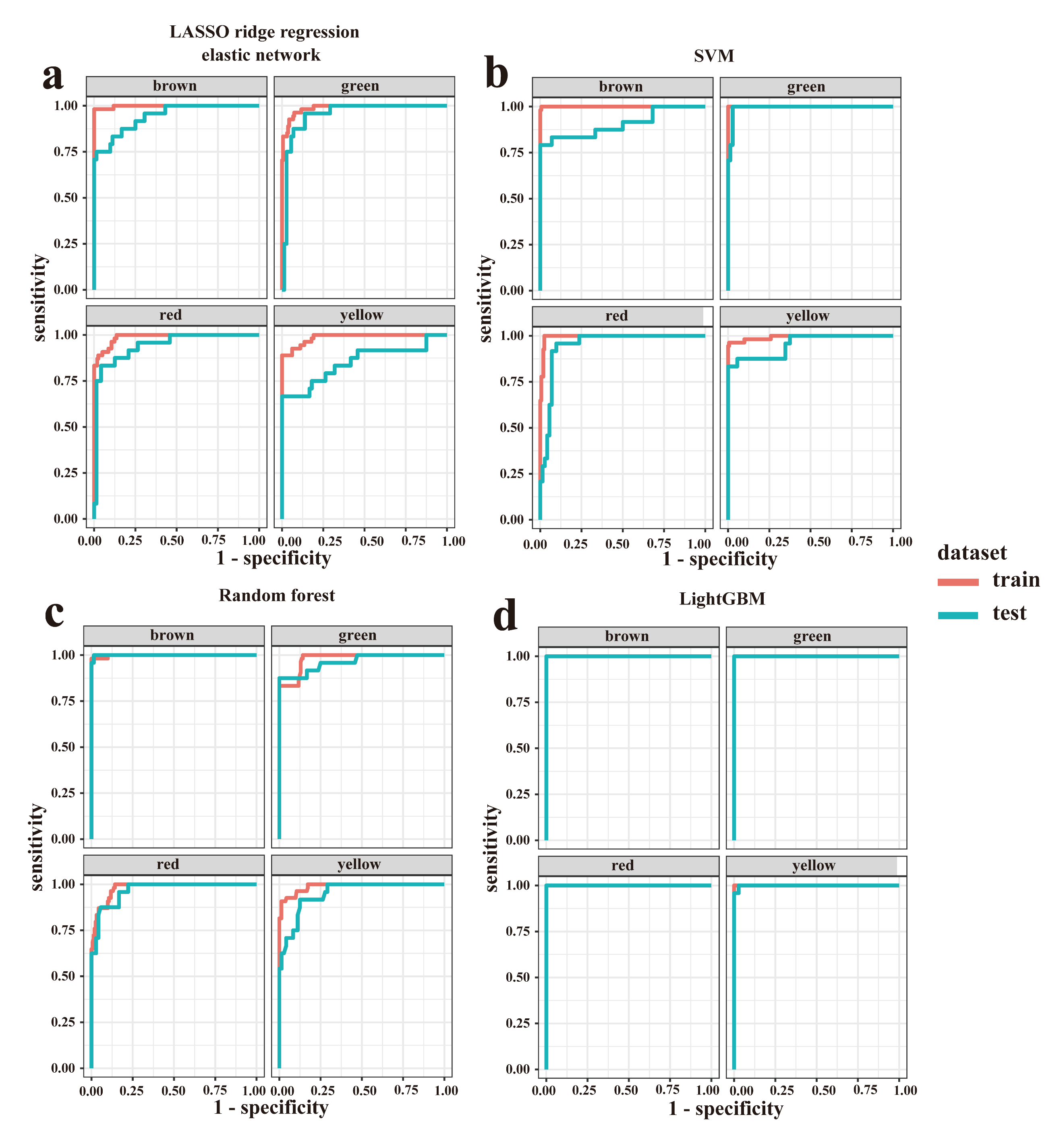
Fig. S3 ROC curves of 4 machine learning classification algorithms.** ROC curves of (a) LASSO ridge regression elastic network, (b) SVM, (c) random forest, and (d) LightGBM algorithms.
